# Anesthesia Practice Shift Scheduling With a Generative Deep Learning Model

**DOI:** 10.7759/cureus.91800

**Published:** 2025-09-07

**Authors:** Wesley Emeneker, Stephen Heape, Gavin Hartman, Mae Gillespie, Stephanie Perkins

**Affiliations:** 1 Anesthesia, PhySS LLC, Fincastle, USA; 2 Anesthesiology, Fraser Health Authority, New Westminster, CAN; 3 Anesthesiology, Reno Tahoe Anesthesia, Reno, USA

**Keywords:** anesthesia scheduling, deep learning, lstm, schedule generation, staff scheduling

## Abstract

Anesthesiology scheduling techniques are inadequate to appropriately deal with modern anesthesia practice demands. Anesthesiologists are increasingly dissatisfied with their jobs in the face of inflexible schedules, increasing workload, and the complexity of practice. Decreasing autonomy and inherent responsibility lead to burnout and decreased job satisfaction. Unfortunately, equitable and timely shift scheduling that meets individual provider expectations remains a distant mirage.

Technology has been promised as a means to decrease workload and improve productivity. But technology has not met these expectations. In real-world anesthesia practice, scheduling remains contentious and time-consuming. These failures are somewhat attributable to current scheduling systems and software. In this paper, we present an alternative method of anesthesiology shift scheduling using advances in machine learning (ML). The development of this deep learning (DL) model for shift scheduling drastically reduces the effort required to create shift schedules that comply with the rules and regulations observed by anesthesia practices. A DL model architecture is developed, trained with shift schedule data from the Reno-Tahoe Anesthesia (RTA) group, and evaluated against the practice requirements. The DL model trained and evaluated demonstrates a Matthews Correlation Coefficient (MCC) of 0.9776 and balanced accuracy of 0.9531. The trained model reliably learns practice scheduling rules sufficient to generate new shift schedules in compliance with the rules. Furthermore, the trained model learns practice rules solely from past examples without requiring a human expert to codify the rules.

## Introduction

Scheduling anesthesiologists is complex and time-consuming due to a variety of extrinsic and intrinsic limitations. Contractual and departmental obligations change over time, and complex shift workload variability exists within any time frame. Predictability is complicated by staffing shortages, difficulties in timely recruiting, leaves of absence, large-scale system stressors, and emergency coverage. Interpersonal factors, such as synchronization of time off and vacation requests, also play a role. Staffing optimization can be controversial due to financial implications. Complexity is further compounded with increasing numbers of facilities and contracts [1,2].

Considering the increasing demands and expansion of anesthesia services, manual scheduling is rarely a viable solution. In this environment, anesthesia departments and health systems have become necessarily reliant on rule-based computing and software. The hope has been that computers and software will simplify the process of scheduling by decreasing the stress, inefficiencies, cost, and time associated with schedule generation [3,4].

Anesthesia scheduling software has inherent limitations. Scheduling software commonly relies on humans to codify the rules of schedule generation and to instruct optimization for the system. For example, a rule may be "no call twice in a row" and an optimization goal may be "equalize call over the quarter". The biggest issue with this codification of rules is that encompassing rules for every situation is too complex to create, much less truly optimize for each situation. Human schedulers regularly encounter special cases and attempt to work around them intuitively without writing down an actual rule or considering all possible solutions to the special case. Then, when a special case causes an exception, the original rule, by definition, is no longer a rule. The ultimate consequence is that rule-based scheduling invariably breaks under expanding complexity or requires expanding time and effort by practice scheduling staff.

Considering the increasingly obvious failures of rule-based scheduling, we have demonstrated an alternative method of scheduling using advances in artificial intelligence. Background on analytical and deep learning techniques is provided, major issues with schedule generation are presented, confounding and limiting factors are detailed, and difficulties with classic mathematical approaches are discussed. Finally, this paper presents a deep-learning (DL) architecture for machine-learned (ML) schedule requirements and demonstrates a real-world case study of ML and machine-generated schedules for an active anesthesia practice.

## Materials and methods

This case study is presented in partnership with the Reno-Tahoe Anesthesia (RTA) group. RTA has 30 different daily shift needs covering multiple facilities. Thirty-one rules and goals guide human schedulers in the creation of short-term and long-term schedules. Examples of schedule rules and goals include: providers should have equal call over six months, providers should not have call the day before vacation begins, any provider on call on Friday will have call the entire weekend, three providers must be on call every weekend, one cardiac provider must be on call each weekend, and a provider should have at most one weekend call per month.

The RTA group provided their scheduling rules and their past schedules for training, testing, and validation of this generative DL model. No protected health information (PHI) or personally identifiable information (PII) was collected or used in this study. The historical schedules used in training and validation are the shift schedules that practice providers actually worked. Additionally, those historical schedules were created based on the schedule rules for the practice.

The dataset provided by RTA is pristine for analysis purposes. The assignments are clear, the rules of assignments are codified, and the data is unaltered. The meanings of each assignment are well-understood, and the intention behind each scheduling rule directly relates to contract requirements, health regulations, and practice needs.

Standard dataset preparation steps recommended by ML practitioners include dataset selection, dataset cleaning and preprocessing, feature selection, data transformation, feature engineering, dimensionality reduction, and labeling. In this study, dataset selection is trivial thanks to a real, complete schedule from the practice with no data missing, incomplete, or extraneous. This is the ideal situation for machine learning because there is no ambiguity in the data. The presented schedule is what actually happened, and the schedule is precisely specified. The model intends to learn patterns and rules for the generation of schedules, so no sampling or dataset reduction is needed. Data cleaning and preprocessing solve problems like missing values, outliers, mis-measurements, and incorrect measurements. These problems have minimal impact on this dataset. Thanks to the quality of the data, the only cleaning required is to fill in the unassigned slots, like weekends off, with the appropriate value.

For preprocessing, we encoded each assignment numerically. An individual practice's assignments may be numbers, letters, full words, or any combination of those variables. Each assignment, including vacation or other time not working, is encoded numerically for ease of use during the embedding and training process. The dataset itself encodes the features, including each assignment, the date of assignment, and the length of the assignment. The model performs transformations by embedding assignments during model training. The layers of the model learn to combine, split, weight, and otherwise recognize which assignments predict others. The learned behaviour is encoded and weighted inside each layer; in essence, the model layers learn to engineer features based on the data seen. No dimensionality reduction is performed on the input data. Each assignment is a single label, and the learning and prediction tasks do not need to be scaled down to fit within a training or prediction context.

We developed and tested a DL architecture and model capable of learning the practice shift scheduling rules of the Reno-Tahoe Anesthesia group. This model was trained on a seven-month tranche and a non-overlapping six-month tranche of the practice’s shift schedule. Within each schedule, an average of 25 provider assignments per day were observed. RTA uses locums (or per diem) and full-time anesthesiologists. Depending on several variables, more or fewer providers may be needed and assigned on any individual day. Actual provider shift schedules from RTA were used for training and testing.

The trained DL model is a combination of the RTA schedule data, the architecture of layers and transformations, and the hyperparameters used to train the model. This model accepts past assignments, remembers previously seen data, weights the assignments from the past, incorporates information from the global schedule, and generates shift schedules that comply with practice rules and requirements. Table 1 lists the generalities the model addresses.

**Table 1:** Generalities of anesthesia shift scheduling CME - continuing medical education

Generality
Not all assignments have hard rules or precedence, but important assignments require hard rules
Practices regularly change the number of providers, or change providers with no change in number
Shifts worked most recently have a larger perceived functional impact than shifts from the distant past
Vacation, CME, and other off time each have different integral patterns in the schedule
A practice’s scheduling rules and goals are learnable from the last few months of the schedule

This simulation study uses a long short-term memory (LSTM) architecture to learn practice requirements from past assignment schedules. Table 2 lists the high-level architecture of layers and the training dataflow of the model.

**Table 2:** Major processing steps for the deep learning model LSTM - long short-term memory

Steps	
1	Embed the input data to derive semantic associations
2	Apply a linear transformation (e.g. $y = xA^T + b$) to the combination of results of the LSTMs, the days of the week, upcoming vacation, and assignment imbalances
3	Apply a LogSoftmax $\texttt{log}(\frac{\texttt{exp}(x_i)}{\sum_{j}\texttt{exp}(x_j)})$ to the results of the linear transformation
4	Calculate the negative log likelihood loss from the LogSoftmax results and the expected results (e.g., the correct assignment)
5	Backpropagate the error using the Adam algorithm to adjust model weights to reduce errors during the next training epoch [5]

Both the DL model and dataset preparation were implemented with PyTorch 2.0. PyTorch implements a DataSet framework to simplify the process of loading, preprocessing, and accessing data for DL training. The datasets used in this case study cover 367 days with 7400+ shift assignments. The number of days included during prediction and training was noted to have a major impact on training convergence, training time, and the effectiveness of prediction. Twenty-one historical days were used for training in this case study. In initial testing, three weeks of assignments achieved a balance between learning the necessary rules without slowing down training. For every 150 days of assignments, each provider has 129 (150-21) different sequences of assignments that can be learned from.

To reduce the likelihood of overfitting, the training data was split into two segments. Eighty-five percent of the data was put into the training dataset, and 15% was put into a test dataset that the model would not see until completion of training. During training, the 85% training set was further split into datasets for training and validation. K-Fold cross-validation was used during training with a 90% training set and 10% validation set.

Back propagation calculated the error and the gradient of the error, and it adjusted the model layer weights to minimize errors. The model was trained for approximately 500 epochs. After each training epoch, 5% of the dataset was uniformly and randomly selected to check the error of the model. If a model failed to improve for 10 consecutive epochs, the previous model was saved, and the training stopped. Batch sizes of 2^6^, 2^5^, 2^4^, and 2^3^ were tested during training.

During the initial training tests, some class imbalances also needed to be addressed. To do this, the following cost-sensitive class weights were tested:

1. Uniform class weights

2. Weight classes as a proportion of the number of data points to the number of classes and the commonness of the class: 

$$\frac{n_{\texttt{samples}}}{n_{\texttt{classes}} \times \texttt{count}_y}$$

3. Weight classes by the natural log of their commonness: 

$$\frac{1}{\text{ln}(\texttt{count}_y)}$$

When uniform class weights were used, the model learned time-off rules well but neglected other assignments. When proportional class weights (#2) and log weights (#3) were used, the model reliably learned assignment and time-off prediction for 29 of the practice rules for training batch size 2^3^. For batch sizes of 2^6^ and 2^5^, training did not produce models capable of predicting rules. For a batch size of 2^4^, training with (#2) and (#3) sometimes produced models capable of predicting rules.

During the development and testing process with cost-sensitive class weights, the authors determined that learning failure with large batches was likely caused by the combination of the regularity of weekends off, the likelihood of a sample requiring the prediction of time-off, and the ease of learning to predict the time-off. To illustrate this point, each 21-day period has three weekends. Most providers will have most weekends off, making the schedule somewhat regular. When 16 or 32 samples are drawn from the schedule for training purposes, it is likely that multiple samples will need Saturday or Sunday assignment predictions. Since Saturday and Sunday are usually free, 1.5 out of every seven samples will be easy to predict (weekend calls and vacations make it less than two out of seven). And since most providers are off most weekends, the model was able to easily learn this variable as a rule.

To test this hypothesis, the dataset was modified to never return more than 1 weekend prediction per batch. With that modification, larger batch sizes were able to learn correct assignment prediction. The modification to the dataset was not made permanent. Instead, restricting the batch size was chosen as the solution to this problem for simplicity and reliability. Over-sampling and under-sampling of the dataset were not tested since cost-sensitive class weights and model training parameter tuning solved the issue of training with class imbalances.

The metrics of Matthews Correlation Coefficient (MCC), balanced accuracy, micro average recall and precision, and micro F1 scores were used to quantitatively evaluate the trained model. The authors performed a qualitative evaluation of generated schedules, determining that the schedules generated by the trained model complied with 29 of the 31 shift scheduling rules of the practice.

## Results

A single, general-purpose scheduling system will not meet the needs of every anesthesia provider and practice. With this core idea in mind, the deep learning model was created to be trainable and usable with low cost and time commitments. Table 3 lists the major time and cost requirements for system use.

**Table 3:** Requirements for construction and use of the deep learning model DL - deep learning

Requirement
The DL model will be trainable for less than $100 of cloud costs
The DL model will be rapidly trainable with modest resources since each practice has different sets of rules and constraints
The DL model can be trained in less than two hours.
The DL model can generate a schedule for less than $1
The DL model can generate 3 months of a schedule for 20 providers in less than 1 minute

Table 4 shows the overarching metrics for all assignment predictions from the training dataset. Table 5 shows the ranges of F1 scores broken out by major types of assignments. These metrics in these tables imply that the model, trained with 7400+ assignments, has successfully learned the rules of staff scheduling sufficiently to make mistakes less than 5% of the time. The presented metrics are generated from the testing dataset of 1000 assignments never used in training. See the Discussion section for more information on how the training and testing datasets were constructed to reduce the likelihood of model overfitting.

**Table 4:** Model metrics Model metrics for deep learning model performance

Metric	Score
Matthews Correlation Coefficient	0.9776
Balanced accuracy	0.9531
Micro average recall, precision	0.9847
Micro F1	0.9446

**Table 5:** Per-class F1 score ranges F1 scores for assignments based on anesthesia-specific assignment importance

Assignment class	Score range
Call	0.9825 - 1.0
Post-call	0.9947 - 1.0
Other assignments	0.9667 - 0.9931

The metrics named in the table have definition variation depending on the specific metric used in different studies, and depending on whether the classification problem is binary or multi-class, so the formulas used to generate the values are given here.

The Matthews Correlation Coefficient (MCC) is defined as: 

$$MCC = \frac{c \times s - \displaystyle\sum_k^K p_k \times t_k}{\sqrt{(s^2 - \displaystyle\sum_k^K p^2_k)(s^2 - \displaystyle\sum_k^K t^2_k)}}$$

Where, $c =$ the total number of elements correctly predicted; $s =$ the total number of elements; $p_k=$ the number of times each assignment was predicted (either correctly or incorrectly); and $t_k=$ the number of time each assignment actually occurred in the real datasethe number of times each assignment actually occurred in the real dataset.

Balanced Accuracy: 

$$\frac{\displaystyle\sum_{k=1}^K \frac{\text{Correctly predicted}_k}{\text{Total assignments}_k}}{\text{Number of classes}}$$

Micro average precision and micro average recall are actually the same metric: 

$$\frac{1}{n}\displaystyle\sum_x \frac{2 \text{Precision}_x \text{Recall}_x}{\text{Precision}_x + \text{Recall}_x}$$

As shown by Opitz and Burst [6], the micro-F1 score - an arithmetic mean over harmonic means - tends to penalize multi-class classification problems with an uneven distribution of bias. Thus, the presented micro-F1 score is the most conservative option, meaning that for the dataset used, it will penalize incorrect answers more than the more common macro-F1 score.

## Discussion

Specification and generation of schedules that satisfy externally-provided requirements has been studied and implemented for hundreds of years. There are a number of techniques used successfully across a range of industries - trucking, construction, airlines, restaurants, universities and schools, and medicine, to name a few. From a mathematical and analytical approach, schedule generation can be solved with logic programming, constraint solving, and optimization, and with convex optimization. Each of these techniques requires that constraints and rules be known ahead of time.

With the amount of effort put into creating good schedules, one might assume the problem is already solved. Interestingly, the problem of scheduling can be solved optimally, but only if some unrealistic assumptions are made. Unrealistic assumptions include ideas like everything will happen exactly as scheduled, all necessary resources are known precisely and ahead of time, no variability in the scheduled resource exists, and each assignable resource is equivalent to every other assignable resource.

In scheduling any complex system, perfect schedules can't exist except after the fact. In other words, perfect schedules don't exist because we can't perfectly predict the future. But the best predictor of the near future is the near past, and we can take advantage of that.

For anesthesia practices and departments, treating schedule generation as a constrained system and solving with constraint optimization techniques or constrained satisfiability are attractive approaches. At first glance, the problems of scheduling staff with a limited number of providers, contract requirements, and practice requirements seem well defined and clear-cut. In practice, provider availability, practice requirements, and even surgery requirements are malleable. For example, a provider taking time off may be available to work if needed, or a surgery centre with a contract for five providers may request an extra provider due to unexpected demand. Additionally, shift schedule rules can contradict each other in uncommon situations. For example, a practice may have rules like "No call immediately before vacation", "No call immediately after vacation", and "No call two days in a row". If the practice has three cardiac-certified providers, we may encounter a situation where those three constraints cannot be satisfied if two of the providers book a vacation at the wrong time. A weakness of constrained solvers is that if an impossible situation occurs, meaning that the constraints of the system are violated, then the solution cannot be completed. The objective optimized function becomes unsolvable and unoptimizable and thus cannot generate results unless other techniques are used to handle these special cases. The potential infeasibility of a solution is a consequence of turning the problem of staff scheduling into a system of equations for solution. There is no single or best way to resolve problems of irreducible infeasibility in these systems; solving these problems requires expert knowledge of both mixed integer linear programming (MILP) and the organization's staff scheduling needs.

Deep learning models don't suffer from the problem of collapsing in the face of contradictory situations. Because DL models learn the probabilities of results based on previously seen data, they can draw on previously experienced information and scenarios to predict the most likely response, similar to human expertise. Deep learning leverages artificial neural networks to autonomously extract hierarchical features from data, enabling machines to recognize patterns, make decisions, and perform tasks without explicit programming [7,8]. The utility of deep learning lies in its ability to analyze complex datasets, discern intricate patterns, and adapt over time, mirroring the nuanced expertise once exclusive to human professionals. Tasks such as image and speech recognition, natural language processing, and complex decision-making in fields like healthcare, finance, and autonomous vehicles benefit from deep learning's capacity to handle intricate information, making it a transformative force in automating processes that traditionally relied on human expertise. The ability of DL models to accurately and precisely learn complex patterns lies in the algorithmic use of error calculation and propagation of corrections when trained with datasets that have answers (aka labels) known beforehand. A DL model is trained by feeding the model dataset samples and correcting its mistakes. When a DL model incorrectly predicts an answer, an optimizer calculates how much error each layer and artificial neuron contributed to the incorrect result. The difference between the correct answer and the predicted answer is called the loss and is dictated by the choice of loss function. The level of contribution (aka weights) is then updated to make a better prediction in the future. By repeatedly doing this process - feed a data sample, calculate how wrong the answer is, correct the model weights to make a better prediction next time - the model will eventually learn a set of weights that is able to make correct predictions for the entire dataset.

Common examples of well-known DL models include convolutional neural networks for computer vision and multi-head attention models like those in GPT4 and Gemini, capable of natural language understanding and generation. These and other large language models (LLMs) mainly refer to neural language models that contain tens to hundreds of billions of parameters, which are pre-trained on trillions of tokens derived from text. LLMs exhibit strong language understanding and generation, and show abilities like learning new tasks from a small sample of examples. The application of LLM to the problem of scheduling staff is largely unexplored. Recent work and surveys show that LLM application to scheduling tends to focus on task planning and scheduling for domains like manufacturing, robotics, and autonomous agents [9,10]. In this context, scheduling relates to the ordering of specific actions. For example, scheduling for a robot might entail closing a gripper on an object, raising the arm, rotating, and then releasing the gripper. This sequence of actions must be correctly scheduled to achieve an objective, but it is a completely different type of scheduling from staff scheduling.

The presented deep learning model architecture avoids some of the issues faced by classic constrained systems solvers for staff scheduling. First, the DL model learned the rules of the schedule from past schedules without requiring a person to codify them. This is a major advantage because a DL model that learns the rules of the system from an existing schedule saves time and energy and requires no expertise in mathematical modeling. Second, the DL model generates results based on previously encountered situations, even in impossible scenarios. The weakness of this approach is that the DL model may generate incorrect schedules or fail to correctly learn all the rules. If a particular scenario or situation is rare, the model may not have enough examples to learn from. A corollary to this is that for the DL model to learn the rules, it must have enough examples. If a specific rule only has one or two examples in the dataset, the model will probably not learn the rule sufficiently for application.

The first stage of the model architecture embeds the assignments, turning names into dense numerical vectors. The embedding process turns a single assignment into a dense vector of numbers that describes the assignment's effects and relationships within the latent space. Roughly, the embeddings will capture the semantic and relational information for each assignment based solely on the previous schedules seen during training. The purpose of creating embeddings is to learn a numerical representation of each assignment so that future stages in the DL pipeline can associate or dissociate assignments to make better predictions. Every assignment has a relationship with every other assignment, some stronger than others. For example, call and post-call have strong influences on each other. The embedding will help learn that relationship, and later layers of the model will use those relationships to learn rules from past schedules. The LSTM layers take the embeddings of the assignments and the matching weekdays of the assignments, and learn how past assignments and sequences affect the next assignment.

**Figure 1:**
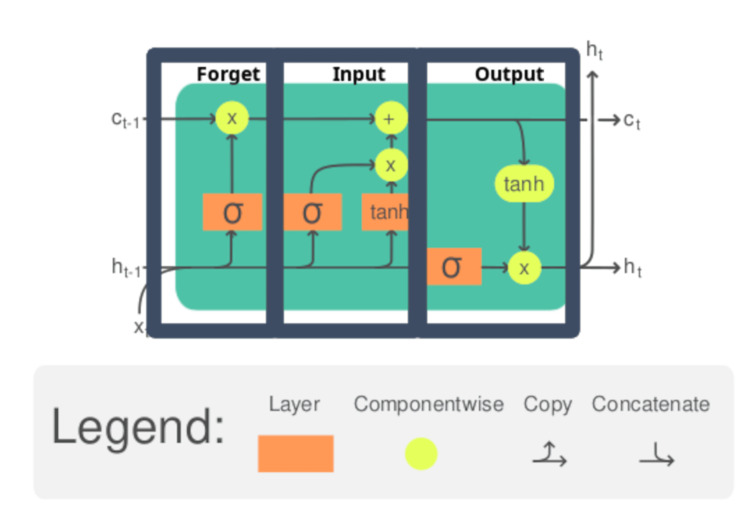
Long short-term memory cell Source: [13]

LSTM is a type of recurrent neural network (RNN) architecture designed to capture long-range dependencies. LSTMs consist of memory cells that store and update information about previously seen data and a set of gates that regulate data flow into and out of the cells. For a complete description of the mechanisms and construction of LSTM cells, see [11,12]. Figure 1 shows a single annotated LSTM cell. Essentially, the cell must determine what to forget, what to learn, and what to pass on for the next timestamp. The cell will take new input and combine it with what it previously knew to determine what to forget, what to learn, and what to pass on to the next timestep. The cell will output the new cell state for the current timestep $c_t$ and the new hidden state $h_t$. As shown in Figure 1, the cell accepts the cell state of the last timestep $c_{t-1}$, the hidden state of the last timestep $h_{t-1}$, and the current input $x_t$.

LSTMs are commonly used in tasks like language modeling, machine translation, sentiment analysis, and sequence prediction. A key feature of each of these problems is that the input data is sequential; the next thing in the sequence depends on the preceding input, and the preceding input loses its influence on the output the farther away it gets. For the generation of shift/assignment schedules, tomorrow's shift for an anesthesia provider depends most heavily on yesterday's shift. Tomorrow's shift depends less heavily on the shifts from two, three, and four days ago. An assignment from 30+ days ago has very little influence on tomorrow's assignment. This work uses LSTMs to both learn practice scheduling rules and to generate schedules in accordance with those rules.

An issue with applying LSTMs to predict the next assignment in a sequence is that a single provider's schedule is not the sole determinant of that provider's next assignment. The past assignments of other providers, the weekday, and known future assignments all influence the next assignment of a single provider. At the other extreme, taking all assignments from every provider causes an exponential increase in data that doesn't increase model performance by the same amount. The solution to this issue depends on the overall goals and needs of the practice. In this study, we incorporate overall counts of assignments versus the mean to help the model learn how to balance things across everyone.

The output from the LSTM layers is passed to a linear layer. The linear transformation makes the data points in the last layer linearly separable before activation with a logsoftmax. The logsoftmax activation ties the model's answers to the negative log likelihood loss (NLLLoss) function used to calculate the error of predicting the wrong assignment. The error between the correct answer (as known from past schedules) and the predicted answer will be backpropagated to update the hidden weights in DL layers and embeddings to make better predictions during future training epochs.

When the training cycle starts, the probability prediction of the next assignment will be wildly wrong. When attempting to learn the rules of a practice with no preconceptions, no previous examples, and completely new data, the initial results will be untethered. As training progresses, the model will learn the correct prediction from the real schedule and will adjust the hidden weights to step the assignment prediction probabilities in the correct direction, improving the prediction and reducing the error.

The objective of DL training is to minimize the error between the predicted value and the correct value. In order to learn how to predict the next assignment, the training process repeatedly makes a prediction, calculates the error from the known answer, and modifies the model weights to make a better prediction next time. A danger to this process occurs when one or more assignments have a drastically higher percentage of the assignments. If assignments A1 and B1 are the highest percentage of assignments, the DL model will optimize for correctly predicting A1 and B1, to the detriment of all other assignments, as long as correct predictions of A1 and B1 minimize the total error. This is known as a class imbalance.

In this case study, the most common assignment is time off - either off for the weekend, or on vacation. Class imbalance is a common problem when DL models are derived from real-world observations. There isn't a single solution that eliminates the problem. Depending on the domain, desired outcomes, importance of prediction, or strictness of answers, it can be addressed in data cleaning, pre-processing, feature engineering, training, or some combination thereof.

Telling the model about the class imbalances may solve the problem. Since we have the full training dataset with labels, we can precisely determine the imbalances of assignments and weight them during the loss calculation and back propagation. This is known as cost-sensitive learning [14]. Another potential solution is to adjust training hyperparameters to minimize the impact of class imbalances. A third potential solution is to over-sample the minority data or undersample the majority data. Artificially increasing the number of less common solutions or artificially decreasing the number of extremely common solutions is another way to reduce the impact of class imbalance [14]. Finally, we may use a combination of cost-sensitive learning and over/under sampling of the data [15] to solve the class imbalance problem.

Another well-known danger when training deep learning models is overfitting. Overfitting occurs when the DL model memorizes the data, unimportant noise, and fluctuations given to it instead of learning the underlying rules. An overfitted model will show excellent scores on all metrics from the training dataset. If the same overfitted model is tested on data it has never seen before, it will almost certainly show poor performance. The result of an overfitted model is that it fails to generalize to unseen data and scenarios. To reduce the likelihood of overfitting, the training data was split into two segments. 85% of the data was put into the training dataset, 15% was put into a test dataset that the model would not see until completion of training. During training, the 85% training set was further split into datasets for training and validation. K-Fold cross-validation was used during training with a 90% training set and 10% validation set.

Related anesthesia-specific scheduling work

A 2023 paper from Sun et al. studies the problem of equitable anesthesia staff scheduling in the face of demand uncertainty [3]. In this work, Sun addresses scheduling variability and equity in the academic department at the University of Texas, San Antonio. The department under study has 50+ anesthesiologists, 50+ residents, and 40+ CRNAs. Sun optimizes the uncertainty and equity in schedules with a multiobjective optimization approach employing mixed integer linear programming (MILP). Sun conducted three surveys among 50+ providers to define 45 workload constraints and objective functions for optimization. Below are a few examples of constraints and objective functions. See the Sun's original paper for definitions of the terms [3].

Defining the maximum and minimum number of shifts assigned to a provider during a scheduling window (equation from sun et al. [3]). $$\begin{split} LowerP_{ph} \leq \sum_{s \in \mathbb{S}} \sum_{d \in \mathbb{D}} A_{psdh} \leq UpperP_{ph}, \\ \forall p \in \mathbb{P}, h \in \mathbb{H} \end{split}$$

Minimizing the maximum two-day average workload (equation from Sun et al. [3]). $$\begin{split} z_2 = \underset{{A,X}}{\text{min}} \underset{p \in \mathbb{P};d,(d+1)\in \mathbb{D}}{\text{max}} \frac{1}{2}\sum_{s\in \mathbb{S}}\sum_{h\in\mathbb{H}}\\ \times(c_{psdh}A_{psdh}\widehat{R}_{dh} \\ + c_{ps(d+1)h}A_{ps(d+1)h}\widehat{R}_{(d+1)h}) \end{split}$$

Minimizing the number of providers required on a given day (equation from Sun et al. [3]). $$ \begin{split} z_3= \underset{{A,X}}{\text{min}}\sum_{p\in\mathbb{P}}pRequire_p \end{split}$$

With careful attention to defining and codifying the problem domain, Sun is able to turn the requirements and desires from the surveys of the anesthesia providers into precisely defined equations suitable for solving by computation [3]. Using the GurobiTM [16] optimizer, Sun generates schedules using the 45 constraints and objective functions for significant improvements in variability in daily workload. Sun also finds that in resource-constrained situations (e.g., not enough extra providers to distribute the load), workload equity tends towards being imbalanced [3].

Eshghali et al. present an integrated model for operating room (OR) scheduling [17]. This work integrates three concepts: mediating elective patients and emergency patients together, considering ORs and downstream units, and proposing hierarchical weekly, daily, and rescheduling models. Eshghali's mathematical model defines 35 equations as constraints and objectives for the purpose of optimizing OR efficiency and improving and bounding patient wait times. Using 20 weeks of surgeries from a hospital in Tehran, combining a predictive model forecasting emergency patient arrival times with a mathematical model for daily, weekly, and rescheduling shows a 10.5% improvement in OR efficiency.

Fügener et al. present two mixed integer linear programming models to address personnel scheduling issues [18]. Fügener breaks down anesthesia provider seniority and specialties for the purpose of call and regular assignments. The ultimate mathematical objective functions are subject to 22 constraints for the duty model (e.g., 24-hour shifts) and 14 constraints for the workstation model (e.g., non-24-hour assignments). Fügener carefully defines the two objective functions that should find the most equitable schedules that meet the contractual obligations, healthcare authority regulations and requirements, and incorporate anesthesia provider preferences. By codifying the scheduling requirements and the regulations and compliance requirements, Fügener describes a system of equations that a MILP solver can use to find schedules optimized for the practice requirements. The objective functions (equations 1 and 24 from ) are reproduced below. For a complete description of the parameters and data definitions, please see the original paper [18]. $$\begin{split} \sum_{j \in J} \sum_{i \in I} \sum_{w \in W} \sum_{t \in T}r^{duty}_{ji}x_{jiwt} -\\ \sum_{j \in J}\sum_{i \in I}\sum_{w \in W}\sum_{t \in T}c^{req-on}\Delta^{req-on}_{jiwt} - \\ \sum_{j \in J}\sum_{w \in W}\sum_{t \in T}c^{req-on}\Delta^{req-off}_{jwt} -\\ \sum_{j \in J}c^{24h}\Delta^{24h}_j \end{split}$$

$$\begin{split} \sum_{j \in J}\sum_{h \in H}\sum_{w \in W}\sum_{t \in T}r^{station}_{jh}y_{jhwt} - \\ \sum_{j \in J}\sum_{h \in H}\sum_{w \in W}\sum_{t \in T}c^{qua}\Delta^{qua}_{jhwt} - \\ \sum_{j \in J}\sum_{w \in W}\sum_{t \in T}c^{dut}\Delta^{dut}_{jwt} - \\ \sum_{j \in J}\sum_{w \in W}\sum_{t \in T}c^{dum}\Delta^{dum}_{hwt} \end{split}$$

Related techniques for shift scheduling

Classic techniques for creating correct, effective staff shift schedules include satisfiability and optimization, convex optimization, and integer and mixed integer linear programming. The Boolean Satisfiability Problem (SAT) is a fundamental problem in computer science and mathematical logic that asks whether a given Boolean formula can be made true by assigning truth values to its variables. SAT is used in logistics, planning, resource allocation, and scheduling. SAT can be used to answer questions like "Given a set of classes with a known number of students, is there a schedule where enough properly-sized rooms allow classes to meet?", and "Given a flight schedule, flight attendants, and FAA regulations, is there a schedule of staff that can crew the flights?". In planning and scheduling, SAT solvers are used to find feasible solutions to complex scheduling problems by encoding the constraints and requirements as Boolean logic formulas. In addition to finding feasible solutions, scheduling often involves optimization objectives, such as minimizing downtime, maximizing resource utilization, or minimizing staffing costs. While SAT solvers cannot directly handle these optimization objectives, complementary techniques like MaxSat can handle optimization objectives [19-22].

Techniques using convex optimization attempt to find the best solution given the constraining parameters in the solution - providers, shifts, contract requirements, etc. The objective of creating a schedule might find many possible solutions. With more than one solution, an objective function that describes how to choose the best solution is required. With different requirements for each practice, there is no single objective function that satisfies the needs of all or even most anesthesia practices [23].

Finally, integer and mixed integer linear programming (MILP) is a third technique that is applied to resource scheduling in other industries. Integer programming deals with constraint optimization problems where both the objective and the constraints are linear and the variables must take on integer values. Shipping and transport companies use integer programming for logistics planning, where the company needs to meet customers' needs with a whole number of warehouses, trucks, and staff. The application of MILP to staff scheduling is especially applicable for optimizing for secondary constraints. For example, equalizing assignments over a period of weeks may need an average of 1.6 first calls per week. By including a non-integer optimization constraint for schedule generation, more equitable schedules can be made. Secondly, framing scheduling problems as an MILP problem makes the solution efficiently solvable. A downside of MILP is that it suffers from difficulties handling non-linearity in constraints and objective functions. Accounting for non-linear effects in schedule generation is an important consideration that any MILP-based solution must address [24,8].

Limitations

A major limitation of this simulation study is the use of data from a single anesthesia practice. Anesthesia practices often have significantly different scheduling rules due to contractual obligations, provider availability, and compensation structure. It is unknown how well the developed DL architecture will learn practice rules and requirements from other practices. While the RTA shift schedules were an ideal choice for the initial work, testing the DL architecture and training process with many other practices will conclusively show if the architecture is generalizable. Training on the schedules of many other practices is the highest-priority future work. In the pursuit of generalization, the authors will analyze how pre-training and fine-tuning may be used to provide faster results and to require less data for training with individual practices. As noted in the limitations, the model failed to learn two of the 31 practice rules. Determination of why the model failed to learn the practice rules is future work. Likely candidates as the source of the problem are too few examples in the datasets, and too much variability in the application of the rules.

## Conclusions

This study shows that a deep learning model can learn the staff scheduling rules and requirements of a practice based solely on historical schedules. Generative deep learning models avoid the inherent biases and singularities of human-created and rules-based schedules. This case also shows that a generative deep learning model can create schedules following rules and requirements without requiring a human expert to codify the rules. As demonstrated in the results section, there are many other obvious advantages of this approach. For example, this model decreases the time required for schedule generation, resulting in lower cost and time saved. It also gives a more accurate representation of the true needs of a practice by accounting for latent variables of practice scheduling, complementing the judgment of human schedulers for special cases, and automatically updating based on the inputted changing needs of the practice.

To the authors' knowledge, this is the first paper demonstrating the use of deep learning as a generative method for medical practice shift schedules. This work improves significantly on shift scheduling research that relies on the mathematical codification of schedule generation rules and goals. The deep learning model presented in this simulation study learns practice scheduling rules directly from past shift schedules. The deep learning architecture required for training is simple to learn, and the computer hardware required is readily available for anesthesia practices and hospital systems. This architecture approaches human-level expertise with significantly reduced cost, faster learning time, and reduced schedule generation time.
